# Survey of microbial populations within Lake Michigan nearshore waters at two Chicago public beaches

**DOI:** 10.1016/j.dib.2015.10.005

**Published:** 2015-10-19

**Authors:** Kema Malki, Katherine Bruder, Catherine Putonti

**Affiliations:** a Department of Biology, Loyola University Chicago, 1032 W Sheridan Rd, Chicago, IL 60660, USA; b Bioinformatics Program, Loyola University Chicago, 1032 W Sheridan Rd, Chicago, IL 60660, USA; c Department of Computer Science, Loyola University Chicago, 820 N Michigan Ave, Chicago, IL 60611, USA

**Keywords:** Microbial community, Metagenomics, Freshwater, Lake Michigan

## Abstract

Lake Michigan is a critical resource for the residents of Chicago, providing drinking water to its 9+ million area residents. Along Chicago׳s 26 miles of public beaches the populous urban environment and this freshwater environment meet. While city-led monitoring initiatives investigate pathogenic bacteria in these nearshore waters, very little is known about other microbial species present. We collected surface water samples from two Chicago public beaches – Montrose Beach and 57th Street Beach – every ten days from June 5 through August 4, 2013 as well as once in early Fall (October 4, 2013). Sixteen bacterial communities in total were surveyed through targeted sequencing of the V4 16S rRNA gene. Taxa were identified using Mothur. Raw sequence data is available via NCBI׳s SRA database (part of BioProject PRJNA245802). OTU calls for each read are also available at our online repository: www.lakemichiganmicrobes.com/bacteria/.

**Specifications Table**

**Table:** 

Subject area	*Biology*
More specific subject area	*Bacterial metagenomics*
Type of data	*Text files: sequences*
How data was acquired	*Illumina MiSeq Desktop Sequencer*
Data format	*Raw*
Experimental factors	*DNA extracted from bacterial cells captured using 0.22 μm filters.*
Experimental features	*Amplification of the V4 region of the 16S rRNA gene. Sequencing using the MiSeq Reagent Kit v2 (500-cycles) kit for the Illumina MiSeq platform.*
Data source location	Chicago, IL, USA: Montrose Beach (41°58′0.71″N 87°38′13.35″W) and 57th Street Beach (41°47′25.54″N 87°34′41.25″W)
Data accessibility	*Raw data is available through NCBI’s BioSample database by following this link. BioSample IDs include: SAMN02739519, SAMN02740899, SAMN02740904, SAMN02740986, SAMN02741333, SAMN02741334, SAMN02741335, SAMN02741346, SAMN02741347, SAMN02786787, SAMN02786788, SAMN02786790, SAMN02786791, SAMN02786793, SAMN02786811, SAMN02786812. OTU calls for each of the reads can be accessed and downloaded at**www.lakemichiganmicrobes.com/bacteria/**.*

**Value of the data**

• While bacterial species known to cause human disease are well studied in the Great Lakes, little is known about other microbial species present.
• The raw metagenome data is publicly available for further analysis and comparison to microbial communities within other urban and rural freshwater environments.
• Sampling regime provides the opportunity to consider temporal and spatial variation between microbial communities within the nearshore waters.

## 1 Experimental design, materials and methods

### 1.1 Sample collection

Two Chicago beaches, Montrose Beach (41°58′0.71″N, 87°38′13.35″W) and 57th Street Beach (41°47′25.54″N, 87°34′41.25″W), were selected as study sites given their relative equal proximity from Chicago׳s city center. The recreational swimming area of Montrose Beach is abutted to the north by the Montrose Beach dog park and to the south by the Montrose Harbor Marina. In contrast, 57th Street Beach is used solely for swimming. (No specific permits or permissions were required for the water samples collected.) Sampling was conducted at both sites within the recreational swimming area. Water was collected from the surface at a distance from the shore such that the water level was approximately knee-deep (~0.5 m deep). Typically, four individual samples (4 L each) were collected within a 5 m area from each site, the exception being July 5 in which only three samples (4 L each) was collected. This process was repeated every ten days from June 5 to August 4, 2013 and again on October 4, 2013.

### 1.2 Bacterial isolation

Isolation of bacterial cells was conducted through filtration. The water was first filtered through sterile 0.45 μm bottle-top cellulose acetate membrane filters (Corning Inc, Corning, NY) to remove plant matter, sand, debris, and eukaryotic cells. The filtrate was then passed through a 0.22 μm polyethersulfone membrane filter (MO BIO Laboratories, Carlsbad, CA) to capture bacterial cells. There were multiple filters used per collection; the amount of water passed through each filter varied depending on the water׳s turbidity. The filters were then stored at −20 °C until extraction.

### 1.3 DNA extraction

DNA was extracted using the MO BIO Laboratories PowerWater® DNA Isolation Kit (Carlsbad, CA). The protocol recommended by the manufacturer was followed with the exception of an additional heat treatment at 65 °C for 10 min prior to initial vortexing. DNA isolated from each of the individual samples for a given collection date/location was pooled together. Concentrations were verified using the Qubit® Fluorometer (Life Technologies, Carlsbad, CA). DNA was stored at −20 °C until sequencing.

### 1.4 16S rRNA amplification

The V4 region of the 16S rRNA sequence was amplified using the primer combination of 5′-TCG TCG GCA GCG TCA GAT GTG TAT AAG AGA CAG GTG CCA GCM GCC GCG GTA A-3′ (forward) and 5′-GTC TCG TGG GCT CGG AGA TGT GTA TAA GAG ACA GGG ACT ACH VGG GTW TCT AAT-3′ (reverse) (Integrated DNA Technologies, Coralville, IA). These primers include the Illumina adapter overhang nucleotide sequences as well as V4-specific sequences producing an amplicon 359 bp in length. This initial PCR reaction was performed as follows: 2 μL of each primer (200ng/μL), 8 μL DNTPs (Promega, Madison, WI) at a 1.25 mmolar/nucleotide concentration, 1 μL of bacterial DNA, 28.5 μL of nuclease free water and the Platinum® Taq (Life Technologies, Carlsbad, CA) components of DNA polymerase (0.5 μL), 10x PCR Rxn Buffer (5 μL), and 50 mM MgCl2 (3 μL). Each reaction was amplified as follows: initial denaturing at 94 °C for 2 min., thirty cycles of 94 °C for 30 s, 55 °C for 30 s, and 72 °C for 1 min, followed by a final extension at 72 °C for 7 min. Amplification was verified via gel electrophoresis in a 1% agarose gel. Negative controls were also run to confirm there was no contamination within the samples as a residual of the reagents or extraction protocol.

### 1.5 Index PCR

To facilitate multiplexing, each PCR product was subsequently amplified again using primers including the Illumina adapter sequences and indexing sequences for subsequent de-multiplexing. These indexing tags have been utilized for numerous metagenomic 16S studies from a variety of environments at the Loyola University Chicago׳s Center for Biomedical Informatics Sequencing facility (Maywood, IL). Each amplified product was again verified via gel electrophoresis. Subsequent DNA preparation – PCR clean-up, library pooling, and sample loading –followed the standard protocols established by Illumina for the MiSeq Benchtop Sequencer [1]. Sequencing was performed using the Illumina MiSeq Benchtop Sequencer (Loyola University Chicago׳s Center for Biomedical Informatics, Maywood, IL). Paired end reads, each 250 nucleotides in length, were produced using the Illumina MiSeq Reagent Kit v2 (500-cycles).

### 1.6 Sequence demultiplexing

Demultiplexing of the sequence data was automated by the Illumina sequencer׳s CASAVA package.

### 1.7 Taxonomic classification

Sequence analysis was conducted using the mothur package [2] following the protocol for sequences generated by the MiSeq platform [3]. The fastq files generated were first assembled into contigs and subsequently filtered using mothur commands to remove contigs containing putative sequencing errors as well as chimeras (uchime). Reads for which the paired-ends could not be assembled were removed from further analysis. Next, the filtered reads were compared against a local copy of the Silva database [4] in order to ascertain the taxonomy of each read; a cutoff threshold (bootstrap) of 80% was used. OTU clustering was performed using mothur׳s cluster.split command, split to the level of Order (taxlevel=4). Batch files were created to streamline the analysis.
